# Endovascular Biopsy of an Inferior Vena Cava Mass: A Case Report

**DOI:** 10.7759/cureus.37546

**Published:** 2023-04-13

**Authors:** Saeed R Mohammed, Dale Maharaj, Shiva Dindyal, Khaleel Ali

**Affiliations:** 1 Department of Clinical Medical Sciences, The University of the West Indies, St. Augustine Campus, Champs Fleurs, TTO; 2 Department of Vascular Surgery, Caribbean Vascular and Vein Clinic, Port of Spain, TTO; 3 Department of Vascular Surgery, Basildon and Thurrock University Hospitals, Mid and South Essex National Health Service (NHS) Foundation Trust, Essex, GBR; 4 Department of Radiology, Caribbean Heart Care, St. Clair Medical Center, Port of Spain, TTO

**Keywords:** vascular oncology, endovascular technique, endovascular biopsy, inferior vena cava, inferior vena cava tumor thrombus

## Abstract

We describe the case of an 82-year-old female referred to the vascular clinic for further evaluation and management of suspected inferior vena cava (IVC) thrombosis. She had previously presented to the general practitioner with a one-week history of vague abdominal pain in the right and left loins. Contrast-enhanced magnetic resonance imaging (MRI) of the abdomen and magnetic resonance angiography/magnetic resonance venography (MRA/MRV) revealed a 10 cm filling defect in the IVC, with the inferior margin of ≈5.8 cm proximal to the aortic bifurcation and its superior margin in the intrahepatic portion of the IVC. The filling defect had a transverse diameter of 2.6 cm and displayed heterogenous enhancement with contrast.

We performed an endovascular biopsy with fluoroscopy (anteroposterior {AP} and lateral views) being utilized throughout the procedure to locate the mass and position the forceps in the tumor bed. The IVC was accessed via the right common femoral vein with a 10F catheter sheath. The sheath was advanced using the Seldinger technique to within ≈1 cm of the mass; then, a biopsy forceps (Micro-Tech single-use 8.5 mm biopsy forceps, Nanjing, China) was inserted, and six tissue samples were obtained.

We report this case to add to the growing evidence that endovascular biopsy of IVC tumors can be performed safely and effectively.

## Introduction

Malignancy of the inferior vena cava (IVC) is rare, with most cases being secondary to renal, liver, testicular, and adrenal tumors [1]. Primary malignancy is even less frequent, accounting for less than one in 100,000 of all adult malignancies, with less than 500 cases described in the literature [2], and almost always involves leiomyosarcoma of the vessel wall. Histopathologic diagnosis is vital to establish whether an IVC tumor is primary or secondary, as these are distinct entities, with unique treatment plans. The histopathologic diagnosis of suspected tumors is most frequently done via percutaneous biopsy, which may be complicated by procedure-related pain [3], of which massive hemorrhage is a rare but significant complication [4]. Here, we present a case of an IVC mass biopsied via a novel endovascular technique, resulting in minimal bleeding and pain.

## Case presentation

An 82-year-old female was referred to the vascular clinic for further evaluation and management of a suspected IVC iliofemoral thrombosis. She had previously presented to the general practitioner with a one-week history of vague abdominal pain in the right and left loins. Initial computed tomography (CT) scan revealed features suggestive of an IVC thrombus with extensive left iliofemoral thrombosis. Upon presentation at the vascular clinic, there were no clinical findings suggestive of IVC or iliofemoral thrombosis.

**Figure 1:**
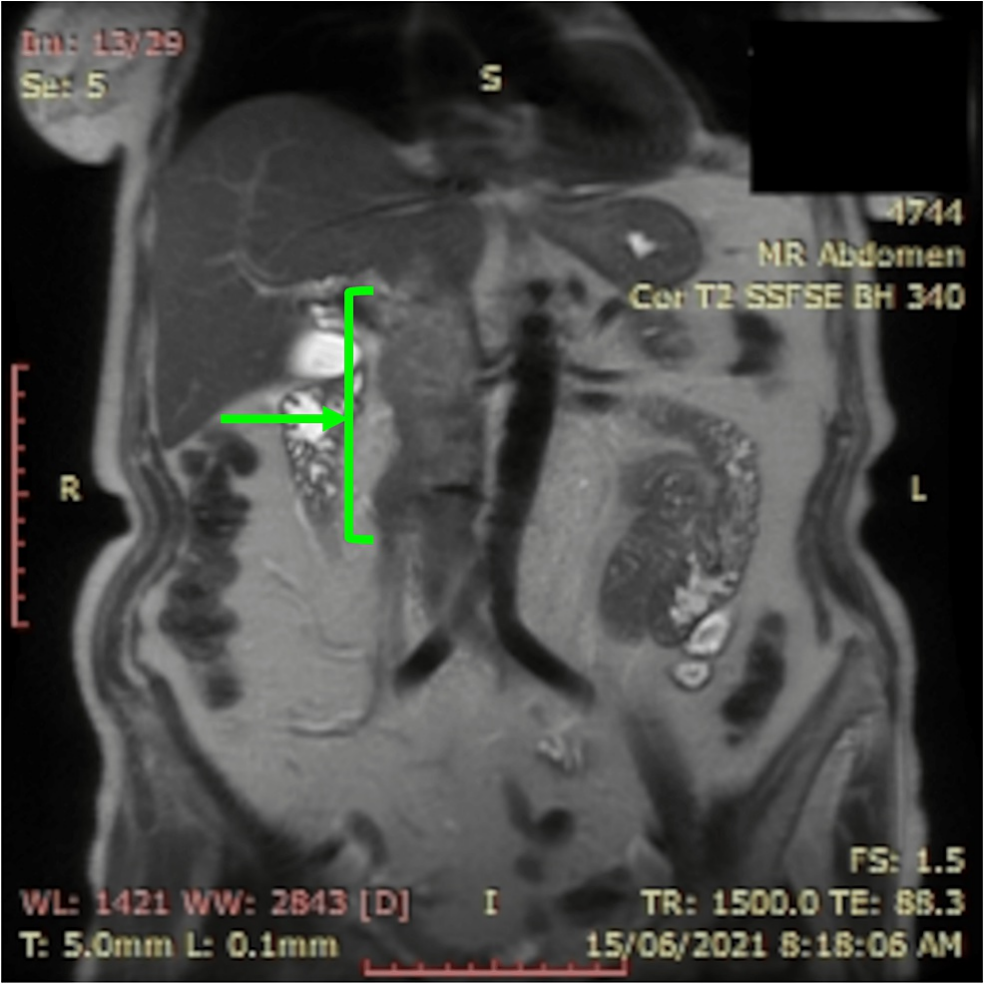
MRI of the abdomen displaying a 10 cm filling defect in the IVC, with the inferior margin of ≈5.8 cm proximal to the aortic bifurcation and its superior margin in the intrahepatic portion of the IVC. MRI, magnetic resonance imaging; IVC, inferior vena cava

A contrast-enhanced magnetic resonance imaging (MRI) of the abdomen and magnetic resonance angiography/magnetic resonance venography (MRA/MRV) were performed, and a 10 cm filling defect was noted in the IVC, with the inferior margin of ≈5.8 cm proximal to the aortic bifurcation and its superior margin in the intrahepatic portion of the IVC (Figure 1).

The filling defect had a transverse diameter of 2.6 cm and displayed heterogenous enhancement with contrast. The kidneys displayed normal cortical parenchyma, and there was homogenous enhancement of the right and left renal veins. The gallbladder, adrenals, pancreas, and spleen were normal, while a small 0.8 cm mildly T2 hyperintense/T1 hypointense lesion was noted posteriorly in the superior right hepatic lobe (segment 7), possibly signifying a metastatic lesion. A low T1/ high T2 signal paracaval mass lesion of ≈2.6 cm × 5 cm was noted, suggestive of infiltrative lymphadenopathy; there appeared to be infiltration of the IVC. It was thus determined that a biopsy of the IVC mass was required.

**Figure 2:**
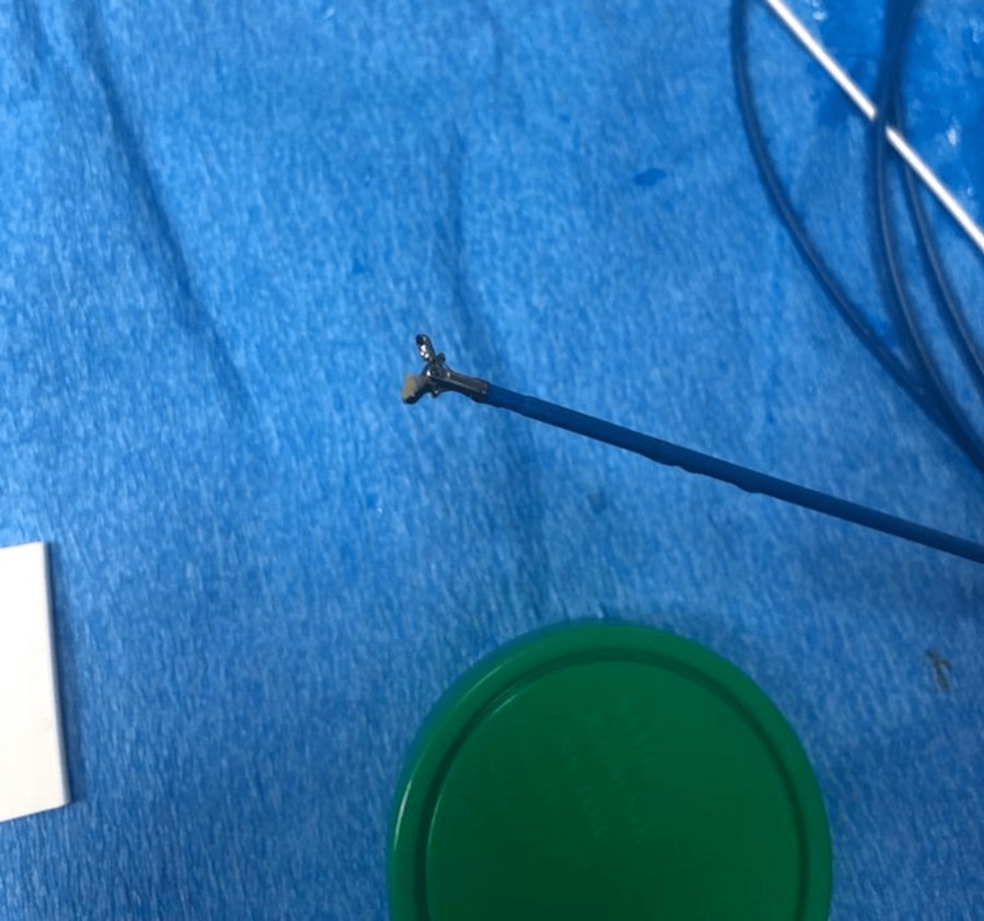
Tissue sample held in a biopsy forceps.

The IVC was accessed through the right common femoral vein with a 10F catheter sheath. The sheath was advanced using the Seldinger technique to within ≈1 cm of the mass. The biopsy forceps (Micro-Tech single-use 8.5 mm biopsy forceps, Nanjing, China) was then inserted, and six tissue samples were taken (Figure 2). Throughout the procedure, fluoroscopy (anteroposterior {AP} and lateral views) was utilized to locate the mass and position the forceps in the tumor bed. No extravasation occurred, and there was adequate tissue for pathology, inclusive of immunohistochemistry.

Microscopy revealed a neoplastic lesion composed of sheets of cells with indistinct borders and round nuclei, many of which contained clear cytoplasm. There was significant tumor cell necrosis. The tumor was strongly positive for cathepsin K and melan-A and positive for smooth muscle actin (SMA) and transcription factor E3 (TFE3) and further displayed focal staining for cytokeratin AE1/AE3 and very weak focal staining for carbonic anhydrase IX (CAIX). The Ki-67 proliferative index was ≈60%. The tumor was negative for desmin, renal cell cancer antigen, human melanoma black 45 (HMB45), cytokeratin CAM5.2, calretinin, sex-determining region Y protein (SRY)-related high mobility group (HMG)-box 10 (SOX10), steroidogenic factor 1 (SF-1), chromogranin, synaptophysin, and inhibin. The overall impression was that of a malignant tumor of predominantly epithelioid morphology, although a definitive sub-classification proved difficult; epithelioid angiomyolipoma and microphthalmia transcription factor (MiT) family translocation-associated renal cell carcinoma were considered the most probable diagnoses. The patient opted against surgical intervention and was thus referred to the oncologic clinic for further management.

## Discussion

The European Society for Medical Oncology (ESMO) recommends that the initial assessment of soft tissue sarcomas comprise radiologic imaging, i.e., either ultrasound, magnetic resonance imaging (MRI), or computed tomography (CT), to determine the extent of tumor involvement, followed by histopathologic diagnosis [5]. Major hemorrhage is a feared complication of percutaneous biopsy, with a reported incidence of ≈0.1%-8.3% in patients undergoing solid organ biopsy [6].

The definitive sub-classification of the reported patient’s tumor proved difficult. The absence of renal mass lesion on radiologic imaging suggested that renal cell carcinoma was unlikely, although there exist reports of metastatic renal cell carcinoma without a primary lesion [7,8].

Endovascular biopsy has emerged as an alternative to percutaneous biopsy [9] and is associated with a lower risk of major hemorrhage [9]. This advantage may be best exemplified in the instance of renal lesions, where endovascular biopsy has been proven to be a safe and effective method of facilitating histopathologic examination in patients who are deemed to be at high risk of bleeding complications from percutaneous biopsy [10].

The efficacy and safety of endovascular biopsy for IVC tumors have thus far been demonstrated primarily in case reports and small case series (Table 1).

**Table 1:** Brief literature review of endovascular biopsy for IVC tumors. IVC, inferior vena cava; CT, computed tomography

Clinical characteristic	Fidias et al., 1997 [11]	Abdel-Aal et al., 2011 [12]	Wei et al., 2014 [13]	Weinberg et al., 2017 [14]	Pomoni et al., 2018 (three cases described) [15]	Kagali et al., 2018 [16]	Balaney et al., 2018 [17]	Massmann et al., 2019 [18]	Lai et al., 2022 [19]
Route of vascular access	Right femoral vein	Right common femoral vein	Right femoral vein	Right internal jugular vein	Common femoral vein	Right internal jugular vein	Right femoral vein	Transjugular	Right common femoral vein
Method used to confirm the location of mass and instruments	Venacavography	CT venogram	Digital subtraction angiography	Fluoroscopy and angiography	Fluoroscopy	Fluoroscopy and IVC venography	Fluoroscopy and IVC venography under digital subtraction angiography	Fluoroscopy	Digital subtraction venography, fluoroscopy, and ultrasound
Method by which mass was approached	An 8F introducer sheath was advanced, and an 8F guiding catheter was used	An 8F sheath was used to access the vein; then, a 5F catheter and 8F guidewire sheath was advanced	An angiography catheter was advanced via the modified Seldinger technique; then, a 6F guiding catheter was used to facilitate suction pressure	Guidewire dilatation	A vascular introducer sheath was used; then, a catheter was advanced	A 10F sheath was advanced into the intrahepatic portion of the IVC via the Seldinger technique; then, a catheter was placed through the sheath and into the proximal intrahepatic IVC	An 8F, 11 cm sheath was introduced, and then, a 6F pigtail catheter was advanced into the lower IVC	Details unavailable	A transjugular guide catheter was advanced through an 8F sheath
Instrument used to facilitate tissue sampling	Biopsy device	Biopsy needle	A 20 ml syringe	A 19-gauge liver biopsy catheter	A 7F biopsy forceps device	Transjugular biopsy needle	An 8F thrombectomy catheter with vacuum-assisted aspiration	Manually shaped angulated 8F aspiration catheter, then endomyocardial straight 6.4F biopsy forceps, and finally off-label directional atherectomy. Only directional atherectomy was successful	Transjugular biopsy needle was unsuccessful. Endocardiac biopsy forceps provided sufficient tissue sample

Although there is little homogeneity in these cases regarding the exact method of endovascular biopsy, it is significant that there were no procedure-associated complications recorded in any instances. Indeed, even if there is bleeding of the vessel wall due to trauma from the various instruments, it would simply traverse the circulation. While further data and larger studies are needed to consolidate the advantages offered by endovascular biopsy compared to percutaneous biopsy and to evaluate if any one endovascular biopsy technique is superior to another, we believe that there is yet sufficient evidence to recommend endovascular biopsy be considered in future patients.

## Conclusions

Malignancy of the inferior vena cava is rare, and a specimen sample is required to establish the diagnosis as either primary or secondary. Endovascular biopsy is an alternative to percutaneous biopsy for the diagnosis of vascular malignancy and is associated with a lower risk of massive hemorrhage. This case adds to the growing evidence that endovascular biopsy of IVC tumors can be performed safely and effectively.
